# IoT-Enabled Smart Cities: Evolution and Outlook

**DOI:** 10.3390/s21134511

**Published:** 2021-06-30

**Authors:** Martin Bauer, Luis Sanchez, JaeSeung Song

**Affiliations:** 1 NEC Laboratories Europe, 69115 Heidelberg, Germany; martin.bauer@neclab.eu; 2 Network Planning and Mobile Communications Lab, University of Cantabria, 39005 Santander, Spain; 3 Department of Computer Security and Convergence Engineering for Intelligent Drones, Sejong University, Seoul 05006, Korea; jssong@sejong.ac.kr

**Keywords:** IoT, Smart City, interoperability, artificial intelligence, standardization

## Abstract

For the last decade the Smart City concept has been under development, fostered by the growing urbanization of the world’s population and the need to handle the challenges that such a scenario raises. During this time many Smart City projects have been executed–some as proof-of-concept, but a growing number resulting in permanent, production-level deployments, improving the operation of the city and the quality of life of its citizens. Thus, Smart Cities are still a highly relevant paradigm which needs further development before it reaches its full potential and provides robust and resilient solutions. In this paper, the focus is set on the Internet of Things (IoT) as an enabling technology for the Smart City. In this sense, the paper reviews the current landscape of IoT-enabled Smart Cities, surveying relevant experiences and city initiatives that have embedded IoT within their city services and how they have generated an impact. The paper discusses the key technologies that have been developed and how they are contributing to the realization of the Smart City. Moreover, it presents some challenges that remain open ahead of us and which are the initiatives and technologies that are under development to tackle them.

## 1. Introduction

Historically, cities and their citizens have spearheaded the enormous technological and social changes that have been taking place continuously, especially since the transition from an agricultural economy to an industrial one [1]. This phenomenon is especially significant from the mid-eighteenth century and it will become more intense if the predictions that forecasts the progressive urbanization of the world population (e.g., around the year 2050, approximately 70% will concentrate in some type of city [2]) finally come true [3]. With these boundary conditions, it is evident that the achievement of more efficient and sustainable cities is an essential objective for which politicians, managers and technicians must work in order to guarantee the quality of life of their citizens [4]. Although this paradigm of sustainability and efficiency has always been important for the managers of cities, it has not been until very recently that technology has made available to the responsible parties a plethora of possibilities that, when properly employed, translate into significant savings [5]. At the same time, the day-to-day improvement of the citizens is consolidating a new urban concept in which the different processes and systems that occur in it are continuously monitored in both time and space [6].

The Internet of Things (IoT) is recognized as a game-changer technology that expands its applicability to a huge variety of scenarios and domains. According to a recent report from McKensey, the potential economic impact of IoT can reach as much as $11.1 trillion per year by 2025, being the biggest source of value of all disruptive technologies, ahead of mobile Internet, knowledge-work automation, cloud computing, and advanced robotics [7]. IoT provides the necessary information to support the context-aware services that are behind the development of smart spaces. With the evolution of IoT technologies, the vision of a Smart City is starting to become a reality. The goal of a Smart City is to establish a wealthy ecosystem where the operation of the city is optimized, increasing its efficiency, but also where new opportunities arise and improve the life of its citizens [8,9]. In contrast to other IoT domains, the Smart City is an agglomeration of many different application areas, including traffic, public transportation, parking, water, energy, waste management, street lighting, public safety, and many more. To support all these application areas, infrastructure has to be deployed in order to enable sensing important parameters of the city, but also for controlling and actuating aspects. The true value of IoT in the Smart City will be achieved if infrastructure is not limited to silos but is made available across different application areas in a homogeneous and resilient manner. The biggest challenges lie in the integration of the heterogeneous underlying IoT technologies and achieving the required scalability and security so that information, even personal and/or critical, can be exchanged among authorized actors and protected from illicit access. In this sense, the key asset is the data that the myriad of sensors embedded in real-world spaces are constantly generating [10]. Moreover, higher level knowledge shall be extracted, giving insights that help in optimizing the operation of the city and quality of life of its citizens. Finally, mechanisms to track the provenance, quality, and value of the information exchanged have to be in place in order to allow trading operations where the raw material is precisely the data that fuel Smart City services.

The contributions of the paper are the following:

1. It gives an overview of the current landscape of IoT-enabled smart cities based on a selection of relevant smart cities initiatives around the world. The number of cities analysed and the depth of the analysis has been intentionally non-exhaustive as the scope of this review is mainly to showcase the different visions and approaches that have been adopted for the development of IoT-enabled Smart Cities around the globe. In this sense, it is important to mention that among the diverse conceptions of smart cities, the paper focuses on those that rely on IoT as a key enabler for realising the smart city paradigm.
2. It describes and analyses the key IoT technologies that have been developed and how they contribute to the realization of a Smart City. The focus of this analysis is on presenting, in a generic and high-level manner, the pool of technologies and services that, apart from the various specific instantiations that have been rolled-out in the existing IoT-enabled smart cities, sets the basic building blocks that are present in today’s IoT-enabled smart cities.
3. It identifies a number of key challenges that are currently being addressed and that are essential for the success of smart cities. This is the key contribution of the paper as it delves into the main issues that have to be addressed in order to overcome the fragmented IoT-enabled smart cities landscape, which can be inferred from the review of cities and technologies made in the paper, and to boost the adoption of a more homogeneous smart city materialization, which eases the roll-out of global smart city solutions.

The remainder of the paper is organized as follows. Section 2 presents a non-exhaustive review of existing cities around the world that can be considered as pioneers and/or front-runner IoT-enabled cities. The section summarizes the solutions adopted, the services offered and establishes the main commonalities and differences between them. The main criterion behind the selection and analysis criteria is the enablement of a comprehensive overview of the evolution of IoT-enabled smart cities throughout the time. In Section 3, the key technologies that have been leveraged for the development of IoT-enabled cities will be analysed, considering the broad challenges that they have addressed. The remaining open challenges that are nowadays attracting the attention of the research and development community as well as those that will do it in the coming years are reviewed in Section 4. Finally, some concluding remarks are highlighted in Section 5.

## 2. IoT-Enabled Smart Cities Landscape

The idea of a smart city first appeared in 1993 when Singapore city presented itself as an “intelligent city”, in [11]. Between 2000 and 2010, the concept of a “digital city” emerged and was closely related to the idea of a smart city. However, it is not until 2010 that the interest in smart cities began to grow exponentially and the first examples of actual smart cities started to be deployed.

In this section we will make a comprehensive yet non-exhaustive review of IoT-enabled smart cities around the world summarizing the solutions adopted and the services offered not only to find commonalities and differences among them but also to try to showcase the evolution that the practical experiences of smart city deployments have undergone throughout the years.

The term smart city is sometimes also used in a broader sense. In some cases, a good communication network infrastructure is already considered sufficient to consider a city to be “smart”, in other cases non-technical KPIs like literacy, organized sports or support for start-ups are considered as key elements of a smart city [12]. In this paper we focus on smart cities that have at least a strong IoT element as part of their smart city definition.

### 2.1. Cities Selection and Analysis Considerations

So far, many city rankings have been published both in scientific literature [13,14,15], as well as by consultancy organizations [16,17]. Thus, this paper is not trying to rank the cities chosen for review. The selection criteria and the features analysed for the cities in the next sections is meant to have a comprehensive yet non-exhaustive list of cities that, in the authors’ opinion, sufficiently represent the evolution of the IoT-enabled smart city concept and, at the same time, exhibit some of the gaps that create the challenges that are still open and need to be addressed.

The list of cities that are reviewed is as follows: Santander (Spain, Section 2.2), Busan (South Korea, Section 2.3), Singapore (Section 2.4), Shenzen (China, Section 2.5), Atlanta (USA, Section 2.6), Amsterdam (Netherlands, Section 2.7), Sunshine Coast (Australia, Section 2.8) and Rio de Janeiro (Brazil, Section 2.9).

As it has been previously mentioned, the intentionally high-level and non-exhaustive review of IoT-enabled smart cities that is presented in the following sections is meant to showcase the fragmented vision and approaches that are provided in cities around the world. In some cases, this fragmentation is present even in the same city. In this sense, the analysis carried out for each city focuses on the idea that, nowadays, commonalities are basically reduced to the offered services, but not on the solutions adopted to provide them neither on the strategies to provide them (not only between one city and another, but also within the same city). Moreover, the selection tries to provide a worldwide perspective, which adds cultural and geo-political variety to the mix, in order to allow generalization of the discussion and the derived conclusions. The objective is to stress the heterogeneity that currently exists in the IoT-enabled smart cities landscape while, at the same time, it is evident that priorities and needs of cities all over the world are significantly similar.

Finally, it is important to mention that, in many cases, the information about actual smart city services provided at field trial or production level (not small-scale or lab-based proof-of-concept implementation), which were the target of the review, is not available through scientific or technical publications but through high-level brochures and/or press releases that hinders stricter methodological analysis.

In summary, the main value of the review and discussion that can be found in the following sections lies on the insights that can be derived in view of the similarity of smart city services that cities of different sizes from four continents have rolled out and the significant differences in the strategy and solutions that they have adopted. The open challenges that are presented and discussed in Section 4 emanate from these insights.

### 2.2. Santander

The SmartSantander project [18] targeted the creation of a European experimental test facility for research and experimentation on architectures, key enabling technologies, services and applications for the IoT in the context of a smart city. The SmartSantander platform includes a continuously growing IoT infrastructure spread throughout the city of Santander (Spain) that currently encompasses more than 12,000 diverse sensors and other IoT devices (fixed and mobile sensor nodes, NFC tags, gateway devices, citizens’ smartphones, etc.).

The deployment, influenced by Santander Municipality’s strategic smart-city service requirements is composed of around 3000 IEEE 802.15.4 devices, 200 devices (mainly gateways) equipped with 3G modules and 2000 joint NFC tag/QR code labels deployed both at static locations (storefronts, tourist-wise points, bus stops, etc.) as well as on-board mobile vehicles (buses, taxis). Moreover, smartphones belonging to citizens who have downloaded related Apps [19] are also part of the testbed infrastructure.

On top of this sensing tier, the platform chosen to manage the management of the underlying infrastructure and the provision of services to end-users and/or application developers is based on FIWARE enablers [20]. Among all the different components of this platform the key one is the Orion Context Broker which exposes NGSIv2 standard interfaces [21] to enable real-time access to observations captured by any of the sensors deployed throughout the city.

Over the deployed testbed and platform, several use cases and pilot services have been implemented:

- **Environmental Monitoring:** IoT devices installed provide measurements of different environmental parameters, such as temperature, CO, noise, or air pollutants. Both static and mobile devices installed on vehicles such as buses and taxis retrieve environmental parameters.
- **Outdoor Parking Management and Guidance:** Parking sensors buried under the asphalt are installed in order to detect parking site availability in downtown outdoor parking zones. Several panels have been installed at the main streets’ intersections in order to guide drivers towards the available free parking lots.
- **Traffic Intensity Monitoring:** Devices deployed at the main entrances to the city of Santander measure the main traffic condition parameters, such as traffic volumes, road occupancy, and vehicle speed or queue length.
- **Parks and gardens irrigation:** In order to make irrigation as efficient as possible, multiple sensors have been deployed in two green zones of the city to monitor irrigation-related parameters and serve this information to gardens’ managers.
- **Waste management:** Paper and cardboard and plastics waste containers in the city are equipped with sensors which are helping to optimize the organization of collection trucks.
- **Participatory sensing:** Users can participate by reporting events or incidences occurring in the city, which will subsequently be propagated to other users who are subscribed to the respective type of events.

### 2.3. Busan

Busan adopted ICT technologies since 2005 under the name of ubiquitous city (u-City). At that time the Busan council focused on the deployment of various sensors to capture valuable data to enhance existing city services. Even at that time u-City used a cloud-based infrastructure delivered by collaboration among participating member companies. However, u-City used proprietary solutions for sensors deployment and cloud platforms. Therefore, devices and platforms used in u-City cannot be reused in other cities. In addition, collected data were stored using non-standardized mechanisms. After 8 years of development of u-City in Busan, the city council learnt that the adoption of global IoT/M2M standards to their smart city can be a key factor to success and develop a sustainable smart city [22].

The Busan smart city, which is the second most populous city in South Korea, initiated its second phase project for the establishment of an open smart city platform based on a global IoT/M2M standards. The second phase of the smart city project was started in 2016 with government funds to achieve interoperability between Service, Platform, Network, Device and Security ecosystem. The smart city introduced seven experimental applications in 2017 as follows:

- **Smart street light**: A service that contributes to energy saving, enhancing street aesthetics, strengthening public safety and crime prevention functions, and building ‘smart lighting’ with energy-saving LED lighting, CCTV, and wireless Internet relay functions.
- **Smart crosswalk:** A service that reduces the number of traffic accidents, and loss rates with a pedestrian detection and car stop detection system to prevent traffic accidents near the crosswalks, thereby reducing social and economic losses caused by traffic accidents.
- **Smart parking:** A service that contributes to the reduction of traffic congestion in the city and the environment by improving the efficiency and convenience of parking so that drivers can check and use empty parking spaces in Busan in real time through mobile app and web service.
- **Building energy management:** A service that establishes an efficient energy saving plan and energy management system through monitoring and analysis of energy consumption by installing a smart meter and sensor in the Busan City Hall.
- **Socially disadvantaged individuals:** A service that delivers the location of the weak people to the guardians and operators based on an IoT specialized network for safety.
- **Lost child prevention:** A service that informs guardians of the location of children in Busan based on an IoT specialized network.
- **Smart store management system:** A service that enables effective store management by monitoring the usage status of various electronic devices used in the store, store environment (temperature, humidity, illumination, fire detection), and visitor trends in real time.

The Busan city council started its third phase smart city project with the concept of data and augmented reality to build a cutting-edge waterfront city through 10 innovative factors such as robots, water, and energy. In particular, it is a city specializing in water by applying smart water management technology in the entire urban water cycle and will also focus on developing a zero-energy system. The third phase of the smart city project also focuses on the development of a data-driven IoT enabled smart city platform.

### 2.4. Singapore

The Smart Nation and Digital Governance Group (SNDGG), which directly reports to the Prime Minister’s Office, oversees the planning and execution of smart city projects [23]. The government operates various smart city R&D projects through many subordinate organizations. Of particular note are the AI-focused ones, including the AI Singapore Project, which is being conducted in collaboration with the National University of Singapore (NUS).

The Singaporean government has established six smart city visions as follows:

- **Smart Urban Mobility:** Uses digital technologies to enhance comfort, convenience and reliability of public transport systems, and support a vision of a car-lite Singapore.
- **Smart Nation Sensor Platform:** This is one of the anchor initiatives in Singapore that enables everyone and everything, everywhere, to be connected all the time in Singapore.
- **National Digital Identity:** A digital identity system for Singapore residents and businesses to transact digitally with the Government and private sector in a convenient and secure manner.
- **Moments of Life:** This is an initiative that aims at providing personalized and pro-active support to citizens at key junctures of their lives.
- **E-Payments:** With this vision, citizens and businesses in Singapore are able to transact digitally in a hassle-free, seamless and secure manner.
- **Core Operations Development and eXchange (CODEX):** This is a digital platform that enables the Government to deliver better digital services to citizens faster and more cost efficiently.

The Singaporean government has developed a policy infrastructure consisting of laws and standardized rules for smart cities, to establish an innovative smart city ecosystem. The IoT Technical Committee was organized to standardize data security and compatibility support that will be applied to various smart city services and infrastructure elements being developed.

To solve numerous urban problems, the Singaporean government is actively helping various actors to utilize smart city data. Key smart city services include MytransportySG that encourages public transportation usage, HealthHub that integrates personal health record management, and Moments of Life that handles birth registration, vaccination, and local event information management on a single platform.

### 2.5. Shenzen

The smart city in Shenzhen, China, aims to strengthen the existing manufacturing industry while connecting it to the advantages offered by the digital industry. The smart city plan established by the city in 2018 includes public services (healthcare, education, community, and weather), city management services (safety, transportation, operation, and water quality), and economic development services (industry, digital economy, and industrial complexes) [24].

Shenzhen is using IoT technology to encourage multiple institutions to share various public services and city management service data. To this end, the city installed surveillance cameras, smart street lights, water quality sensors, and other devices to be connected to a cloud-based IoT platform. Such data are being collected and managed on a city-wide level.

The following describes some of the smart city services operated in Shenzhen.

- **Water quality management:** An IoT platform called Smart Sponge collects and manages water quality-related data on a real-time basis to provide city-wide water management services (water volume, quality information, and location information of water pipe networks).
- **Smart industrial complexes:** This service connects a series of smart factories to share various technologies and provide effective smart factory services. To achieve this, the city established ultra-high-speed broadband Internet, next-generation wireless network, and free Wi-Fi infrastructure, in addition to connecting and managing various IoT devices in industrial complexes via a common cloud platform.
- **Public safety:** The smart public safety service in Shenzhen deals with public safety, production safety, food and pharmaceutical safety, and geological risks in the city. This is achieved through a surveillance camera infrastructure and a network that covers the entire city. This IoT public safety platform was designed to be used all-year-round, and it is used to provide a variety of services including social safety, anti-terrorism and crime prevention efforts.

Key smart city technologies used in Shenzhen include IoT, AI, and cloud computing. That is, IoT technologies help manage various sensors and infrastructure installed in the city. Data are transferred to the cloud-based IoT platform to be collected and shared in real-time. Such shared data are processed using AI technology to provide various intelligent smart city services.

The following are the smart city technologies used in Shenzhen.

- IoT: Video cameras with network features, smart street lights, smart traffic lights, smart water quality management, utility management, environmental pollution management, smart meter, smart factory, etc.
- Big data and AI: Social credit platform, intelligent transportation system, fire incident analysis system, smart surveillance camera, intelligent parking service, and smart maintenance service.
- Cloud computing: Cloud-based IoT platform and city data management centre

### 2.6. Atlanta

The City of Atlanta defined three main areas, namely mobility, public safety and sustainability on which its smart city plans would be broadly focused. The decision made was to employ a data-centric model that exploits data collected from a wide variety of sensors and existing information sources and enables the adoption of better-informed decisions in order to enhance the effectiveness of city operations and have a positive impact on citizens’ and visitors’ lives. Overall, the idea was to horizontally integrate city departments through data sharing as well as increase interaction with citizens by publishing data openly. By using ICT technologies and analytics it is looking for automating city processes and improving decision making by leveraging data to gain situation awareness, service optimization and predictive analytics.

Some of the projects, with a focus on those more related with ICT technologies in general and IoT in particular, that have been put in practice in Atlanta are as follows:

- **North Avenue Smart Corridor:** Designed to act as a living lab for mobility- and safety- related IoT and data analytics, as well as connected and autonomous vehicles. The 2.3-mile North Avenue Smart Corridor, which is a key east-west arterial connection in Atlanta, incorporates many smart city technologies, including adaptive traffic signals which adjust to traffic conditions in real-time and can prioritise emergency vehicles. The City of Atlanta will take key learnings from this Corridor but is now looking to take a much broader approach to mobility.
- **ShotSpotter:** This service can pinpoint the exact location where a gunshot has been fired. Alerts will notify police of possible gunfire within 30–45 s, and give the precise location, normally within 20 feet. The necessary infrastructure was deployed at 200 streetlights in five different locations across Atlanta.
- **Smart Neighbourhood:** The project, run in association of an energy utility and a home construction company, planned the creation of more than forty technology-enhanced houses aiming at drastically reducing their Home Energy Rating System (HERS) score. The data-fuelled home energy optimisation platform intelligently scheduled each home’s major appliances, in coordination with solar and batteries, to minimise cost while maximising each homeowner’s comfort at a neighbourhood level.
- **ATL311 and NotifyATL app:** These two apps enabled more fluent interaction with citizens which could report non-critical problems such as potholes, graffiti or waste issues and track the status of service requests, as well as provide citizens with critical information about events such as severe weather, unexpected road closures, missing persons and evacuations of buildings or neighbourhoods as well as community events and crime alerts.

Most of these projects are supported by CityIQ platform [25]. This IoT platform, currently run by Ubicquia, was developed by Current (a General Electric subsidiary) and built on Intel’s technology and was used to make the available data actionable to support the abovementioned services.

### 2.7. Amsterdam

Amsterdam is taking a bottom-up approach to developing a smart city. A public-private partnership with more than 600 partner organizations has been set up. The idea is that rather than being technology-centred or city government-driven, the citizens and the value that smart city solutions bring to them should be the focus [26]. Some high-level goals have been defined, like *sustainable economic growth*, *efficient use of natural resources* and *high quality of life*. In general, the smart city approach has a “think globally, act locally” angle [27].

The following describes a selection of projects that are or have been conducted as part of “Amsterdam Smart City”, with a focus on those that have a relation to IoT technologies [28,29]

- **Open Data:** Availability of open data from about 30 city departments, including topographical and address data, land value and ownership information, healthcare data, traffic data and more. In most cases the open data is provided as Excel sheets accessible from a web site, i.e., easily accessible to humans, but not directly linkable and automatically accessible to machines.
- **MyNeighbour App (MijnBuur):** provides a direct connection between neighbours, e.g., for the purpose of alerting about dangers and identifying something that needs to be done. The idea is to strengthen social responsibility and direct interactions between citizens without having to involve the municipality.
- **Wyzer App:** implements the idea of keeping people off the main track, thus reducing congestion and improving their city experience at the same time. With fuzzy navigation, they are directed in the right direction, but not on the main route. The application highlights “hidden gems” on the way.
- **Plastic free rivers:** is a pilot for stopping plastic floating on rivers. A tube with holes is placed at the bottom of a river and air is pumped through it. This creates a “bubble barrier” that stops floating plastic and guides it to the river banks for collection.
- **Social Glass:** does big data analysis on social media and, on this basis, determines the emotions of the public. This information is cross-referenced with geolocation and other data, which enables establishing patterns and mapping the mood of the city.
- **IoT Living Lab:** An IoT Living Lab has been set up, covering a stretch of more than 3 km with iBeacons using LoRaWan technology. Users can use it to send small data packets to the cloud, which can then be accessed and used for smart city apps.

### 2.8. Sunshine Coast Region

The Sunshine Coast Region in Australia has started to implement a smart city program in 2016 with the goal to “build a stronger economy and a safer community, and improve service delivery to our residents, businesses and visitors.” [30]. The expected benefits include reduced carbon emissions, traffic congestion and consumption of resources, improved standard of services and quality of lifestyle for residents, reduced costs of service delivery, increased safety and data-driven design and planning. The Smart City Framework was developed together with Cisco and Telstra.

Specific projects that have been implemented are the following [31]:

- **Sunshine Coast Council (SCC) App:** provides information about facilities, events, guided tours, but also serves as a disaster hub with emergency contacts.
- **Smart Street:** with free public WiFi collecting data to understand how public spaces are used. Overall, more than 200 access points have been deployed.
- **Pedestrian and cyclist counters:** monitoring how many people use public areas like parks and walking trails, allowing better planning, maintenance and cleaning.
- **Networked LED street lighting:** is turned on and off automatically during twilight periods to reduce energy consumption and thus electricity costs and CO_2_ emissions.
- **Waste bin sensors:** to measure waste levels and send alerts if the bin is full or still empty to help deliver more cost-effective waste management services.
- **Networked irrigation system:** monitors the soil and sends alerts when watering is required. Additionally, flow meters identify problems and leaks to reduce the waste of water.
- **Networked flood sensors:** installed on roads and bridges, which are susceptible to flooding.

### 2.9. Rio de Janeiro

Rio’s Smart City strategy was originally bound to the celebration of the Olympics in 2016, but a fatal landslide in 2010 accelerated the construction of a Centre of Operations to tackle natural disasters and coordinate relevant emergency responses. The solution adopted was based on IBM’s proprietary platform for smart cities. The initiative is already largely centred on safety and security, both in terms of disaster prevention and management, and freedom of information.

Regarding the main services that the city is providing, they are as follows:

- **Emergency monitoring and response.** The video feeds from the surveillance cameras installed in the city are the core of the Intelligent Operations Centre (IOC) focusing on controlling traffic and weather to ensure smooth functioning of day-to-day operations.
- **Local government data sharing.** Departments from the local government hosted at the IOC premises exchange data amongst themselves using IBM’s platform to increase the efficiency of their services.
- **Rio Agora.** A platform which combines an online social platform with the already existing portal called Central 1746 [32] which allows citizens to propose and debate public policy with municipal departments and agencies in different themes as well as to request a number of services from the city’s government such as waste removal, repair of damaged roads and walkways or reporting of illegal activity, the requests posted on the website are integrated in the IOC and its lifecycle managed through the system.

### 2.10. Cities Review Summary

As it is summarized in Table 1, when looking at the cities, it becomes clear that there is significant heterogeneity. No two cities are the same, nevertheless there are also commonalities. The high-level vision of cities typically includes *achieving a better understanding of the city* with the objective of *improving the city*, in particular *public services*. Usually, general goals are to *improve safety and security*, *increase energy-efficiency*, *reduce the use of resources*, *achieve sustainability*, *strengthen the economy and improve the communication*, between the city and its citizens, but also among the citizens. The strategy to achieve this includes collecting information through sensors, cameras, but also direct input from the citizens. An important aspect is also the improvement of the communication infrastructure and the computing backend and, overall, the digitization of important processes. Whereas in some cases, services are immediately rolled out on a large scale, often there are smaller scale pilot projects first, to test and gain experiences with services. In general, a city does not become “smart” in a single step, but it is an evolution with multiple phases. The early adopter cities have started to develop smart city solutions around 2010 and most of the selected ones have already worked on becoming a smart city for a few years.

All the IoT platforms of the cities are different; only two are based on standards, the others are proprietary solutions, mostly developed by one or more big IT companies. Different wireless technologies are used, the most popular ones are cellular, WiFi and LoRa and often more than one of these technologies is used. The deployment of sensors, cameras and base stations differ very much, both with respect to the technology used and the size of the deployment. All cities have implemented a selection of different applications and services. The most popular ones are in the area of traffic, parking and water, but also waste, lighting, safety and participatory sensing can be found in multiple cities. However, there are also applications that are specific to single smart cities like the fuzzy navigation highlighting hidden gems or the happiness meter service.

Table 1 summarizes the key features that have been analysed from the reviewed cities from around the world.

## 3. Key Technologies Developed

In a smart city, various sensors and actuators are deployed to measure useful data around the city. Such IoT devices exchange messages (i.e., measured data and commands to actuators) to the IoT platform through various network technologies. Stored data on the IoT platform for smart cities is used in smart city applications to resolve various problems in the city. In recent years, intelligent smart city services using artificial intelligence and big data technologies have begun to be introduced. As data security and privacy are becoming important, related technologies have begun to be applied as essential aspects to smart city platforms. Therefore, in this section, we provide an overview of the main technologies (incl. standards) for IoT devices, networks, platforms, artificial intelligence & big data and security.

### 3.1. Sensor and Actuator Technologies

The IoT is formed by multiple technology tiers. They permit sharing of information among things over the Internet to eventually deliver smart, self-sufficient actions, the value of which heavily relies on the quality of the information itself. Therefore, sensors and actuators are a crucial slice of the IoT technology stack and a fundamental aspect in the deployment of every IoT system.

Sensors are the devices dedicated to detecting events or changes in their surroundings and transform these physical phenomena (e.g., temperature, noise, air humidity, pressure, presence of substances, etc.) into voltage and/or current signals that can be correspondingly understood. On the other hand, an actuator, is a device that converts the commands that it receives in the form of electrical impulses into any kind of mechanical motion which alters its close environment. This is done through a range of simple actions typically including, but not limited, to opening and closing valves, activating or deactivating relays, changing their behaviour (i.e., colour, position, etc.) or emitting some kind of alarm.

Such devices have been around for many years already. They are tightly bound to industrial applications of any kind. However, their ability to be miniaturized and wirelessly connected through the Internet has created an unprecedented explosion of new opportunities and applications of this kind of devices in a huge variety of application domains, including smart cities as can be seen from the examples described in Section 2. The IoT has simply transformed the way in which they are used as it enables that the data that they generate can be consumed by cloud-based data analytics to develop intelligent solutions for machines, people and environment alike.

They form the baseline of IoT and it is critical to have efficient and low-cost sensing and actuating solutions as they are ultimately responsible for monitoring and controlling the environment, and streamline operations in almost every kind of sector, from smart cities to environment protection.

There are many different types of sensors and actuators in an IoT system. Flow sensors, temperature sensors, voltage sensors, humidity sensors, and the list goes on as the application scenarios do (cf. Section 3.4). In addition, there are multiple ways to measure the same thing.

However, there is one fundamental issue that can broadly characterize the design of smart city monitoring systems, namely, the way sensors and actuators are deployed. In this respect, it is possible to differentiate between stationary sensors, mobile sensors and crowd-sourced sensors.

- **Stationary sensors:** In the static deployment of sensors, all the nodes are stationary. In this case, the most important aspect is the design of the network, i.e., specifying beforehand where the sensors should be deployed for an optimal monitoring, paying special attention to providing a good coverage of the monitored area and to guaranteeing connectivity of the wireless nodes. Static WSNs are heavily used in smart city scenarios as they are well-suited for smart metering of public services networks such as water and sewage [33,34], public lighting [35], parking [36] or traffic monitoring [37,38,39], as well as for generating the baseline monitoring of the city environment [38,39].
- **Mobile sensors:** In the case of mobile deployments, nodes are installed in vehicles that move around to collect data. These vehicles could be specially designed robots [40], or more recently drones [41], or public vehicles that are equipped with specific sensors [42,43]. Mobile sensors are a sensible solution to enhance the performance of city monitoring in terms of coverage, cost or resiliency, to mention a few. In this sense, they are mainly used to complement the sensing of existing static WSNs. For example, while city traffic monitoring is typically addressed using road side cameras [44] and inductive loops [45] embedded in the road, many cities are using the information collected from sensors in vehicles, usually public vehicles like buses [46] or garbage trucks [47] to have more detailed and fine-grained information. Additionally, some aspects like road surface conditions cannot be correctly monitored through stationary sensors. On the downside, mobile nodes introduce new challenges and problems, mainly related to the connectivity guarantee. Additionally, for the design of the network, it is important to consider the paths that the vehicles will follow in order to consider how well the sensors they are equipped with will cover the monitored area.
- **Crowd-sourced sensors:** Crowd sensing basically means outsourcing the monitoring of the environment to the crowd. In cities, this mainly relates to the capacity of leveraging the abundance of digital devices that are equipped with sensors. Citizens’ smart phones are the most commonly used ones, and also the ones providing rich information. Crowd sensing is typically catalogued into two main types: participatory sensing [48,49] and opportunistic sensing [50,51]. In participatory sensing, the users are directly involved in the sensing action. For example, in [52] citizens reported issues like broken public infrastructures (e.g., streetlights, benches, bins, etc.) or cultural activities (e.g., street art, festivals, etc.). In opportunistic sensing, the users might be unaware of the sensing of the smart phone. For example, the solution presented in [53] is designed to monitor the urban traffic and road surface conditions and an estimation of air quality and PM2.5 in cities is presented in [54].

### 3.2. Networking Technologies

The IoT environment consists of an enormous number of smart devices, but with many constraints. Reduced processing capability, storage volume or available energy are among these constraints. Therefore, the IoT implementation requires communication protocols that can efficiently manage these conditions.

As far as wireless IoT is concerned, many different wireless communication technologies and protocols have been used to connect the IoT devices. They can be coarsely catalogued into two main groups, namely Wireless Personal Area Networks (WPAN) and Low Power Wide Area Network (LPWAN), both of which can be found in the smart city deployments described in Section 2.

The first ones are characterized by supporting short range communications with minimum complexity and power consumption while keeping medium/low data rates. IoT devices equipped with these technologies can establish centralized networks but, typically, they are arranged in multi-hop ad-hoc networks so that they can be deployed in relatively large areas. These kinds of technologies were the ones that were firstly available and monopolized the pioneering IoT-enabled smart city deployments. The most used technologies falling in this category are specified by the IEEE 802.15.4 Working Group and Bluetooth Special Interest Group (SIG) respectively.

The IEEE 802.15.4 standard defines the lower-layers’ communications protocols (i.e., PHY and MAC) for low-data-rate wireless connectivity with fixed, portable, and moving devices with no battery or very limited battery consumption requirements. Many existing IoT deployments employ Internet Protocol Version 6 (IPv6), over Low power Wireless Personal Area Networks (6LoWPAN) [55] to set up the network among IoT devices equipped with IEEE 802.15.4 interfaces and connect them to the Internet. However, the majority of cases uses a clustered approach where IEEE 802.15.4 IoT devices create a network among themselves and include in it a gateway that is used as sink of all the information generated by the IoT devices in the cluster. The gateway then proxies that information to the Internet. While there are some proprietary solutions following this architecture like DigiMesh [56] or Z-Wave [57], the most well-known one is Zigbee [58], which uses IEEE 802.15.4 as a baseline and defines networking and service provisioning protocols on top of it.

Other technologies like Bluetooth [59,60] or and NFC [61] have been also considered and largely used as solutions for enabling IoT-enabled spaces in general, and smart cities, in particular. Concerning Bluetooth, the profile that has been mainly used for IoT environments is the Bluetooth Low Energy (BLE). The advantages of BLE include lower power consumption, lower setup time, and supporting star network topology with an unlimited number of nodes thus providing networking capacity that is well-suited for IoT scenarios. Both RFID and NFC use similar technology principles enabling very short-range communication technologies that enable data transmission among devices touching each other or being within a distance of no more than a few centimetres. Besides its use for instrumenting Points of Interest with unique information that can be retrieved by the reader if it comes close enough, NFC enables the commissioning and control of IoT devices.

Pertaining to the second category, the LPWAN technologies, they appeared much more recently, but they have quickly gained momentum and the current trend in IoT deployments for smart city scenarios is to use this kind of communication protocol. They provide the main features demanded by technologies for IoT, namely low-power consumption and low-complexity, but provide much larger coverage (in the range of hundreds of meters to kilometres) and the capacity for supporting hundreds, or even thousands, of nodes in one network. In its basic mode, which is the one typically used in the existing deployments, they all share a star-like topology where a base station serves all the IoT devices under its area of coverage. On the downside, the data rate supported by these technologies is significantly lower than their WPAN counterparts. The most widely spread technologies in this category are LoRa [62], SigFox [63], NB-IoT [64] and LTE-M [65]. Each has their respective advantages in terms of different IoT factors [66]

Sigfox was developed and is operated by the same company, which builds the communication modules and operates its own IoT solution in 31 countries and is still under rollout worldwide [67]. LoRa was also first developed by a microelectronics company but has been already standardized by LoRa-Alliance and is deployed in 42 countries and is still under rollout in other countries owing to the investment of various mobile operators. Finally, NB-IoT is based on narrow band radio technology and is standardized by the 3rd generation partnership project (3GPP). Its specifications were published in Release 13 of the 3GPP on June 2016. Like Sigfox, LoRa uses unlicensed ISM bands while NB-IoT works on LTE licensed frequency bands. As it has been said, the three of them supports coverage ranges of kilometres even in urban environments and can allow several years of battery-powered operation thanks to extremely reduced consumption. However, they are limited in data rate (from the maximum 100 bps of Sigfox to the 50 kbps of LoRa or 200 kbps of NB-IoT) and payload size (12 bytes in SigFox or 243 bytes in LoRa). Another important difference among them is their deployment model. While SigFox and NB-IoT networks are rolled-out by licensed network operators only, LoRa can support privately owned networks since both the wireless technology and the network infrastructure specifications are openly available.

In summary, there is not one single solution that fits all cases. Instead, IoT-enabled smart city deployments have made use of the wireless communication technologies available at the moment the deployment took place which best covered their requirements. In general, WPAN technologies were mainly used by the first IoT deployments as they were the only solution available, while nowadays LPWAN solutions are the most common choice. Future 5G networks are called to pick up the baton as they will not only support the low-power, wide coverage and scalability requirements but provide enhanced communication characteristics, such as reliability, quality of service and/or security, which are necessary to fulfil the communication requirements of some smart city services for which current communication technologies do not guarantee the required grade of service.

Table 2 shows the key features of the above mentioned IoT network technologies.

### 3.3. IoT Platforms

Most existing commercial IoT platforms enable the user to connect devices to a vendor-operated cloud. Sensors send their data to the cloud for storage and analysis and actuators receive commands from there [68,69,70]. Some device vendors provide IoT clouds for a specific device technology or even a specific brand, whereas established general cloud providers typically support a wider range of technologies in particular based on the networking technologies described in the previous section. The tools provided by the vendor make it easy to integrate and manage the devices. In addition, there is support for some analytics and the visualization of the IoT information and the status of the deployment. The business model is targeted towards binding the user to the respective vendor, with the idea of selling more cloud resources, services and tools, possibly also additional devices. In the best case, a significant effort would be required to switch to a different platform.

While such a business model may still be attractive for private users or for smaller scale commercial deployments, the resulting vendor lock-in poses a significant risk to a smart city. A smart city may need to integrate heterogeneous existing deployments, has to be able to scale up deployments over time and possibly integrate external stakeholders that may also have existing deployments.

Another aspect is that there are technological developments moving IoT information processing to the edge, reducing bandwidth requirements, e.g., for processing video streams, and reducing latency and thus enabling control loops with hard real-time requirements. While some providers are in a position to provide edge computing facilities, e.g., a network provider that can extend existing deployments with edge computing resources, other providers are not in this position and may also not be able to easily integrate user-deployed edge resources.

To reduce the risk of vendor lock-in and of being barred from technological developments as in the case of edge computing, smart cities are looking towards standards, for whole platforms or for specific functionalities and interfaces, e.g., for managing devices or requesting information. If complete deployments are based on standards, switching platform providers and integrating different deployments become feasible.

In the following we look at some existing IoT standards and shortly describe what aspects they cover:

- **oneM2M** standardizes an IoT service layer that constitutes software middleware between IoT hardware and communication technologies on one side and IoT applications on the other side. oneM2M [71] is a partnership project, whose partners are the regional telecommunication standardization organizations in Europe, Asia and North America, and whose respective member organizations drive the standardization process. The oneM2M service layer provides common service functions for data management, security, device management, connectivity and group management. A REST-style API provides standardized mechanisms for building REST resource structures and supports standardized interaction patterns. For the abstract API, different communication protocol bindings have been defined, including HTTP, CoAP and MQTT. oneM2M is agnostic to the IoT information model used, i.e., it can store and handle any type of IoT information.
- **OMA Lightweight M2M (LwM2M)** is a protocol for IoT device management and service enablement. It is standardized by the Open Mobile Alliance (OMA) [72]. LwM2M defines and application layer communication protocol between a LwM2M server and a LwM2M client located on each device. The device management functionalities are connectivity management, provisioning of security credentials, firmware updates and remote device diagnostics. The service enablement functionalities are sensor and meter readings, actuation and configuration of devices.
- **NGSI-LD** provides a Context Information Management API and an underlying linked data-based information model resulting in a knowledge graph. NGSI-LD is specified by the Industry Specification Group on cross-cutting Context Information Management of the European Telecommunications Standards Institute (ETSI ISG CIM) [73]. It represents the evolution of the NGSI context interfaces originally standardized in OMA and later evolved in the FIWARE open-source ecosystem. Applications can manage, retrieve and discover the information they need, using filters and geographic scopes to limit the results to what is relevant. Using NGSI-LD, applications get information on a suitable abstraction level and independent of device technology specific data models.

To help cities in building future-proof and interoperable smart city infrastructures, there are city networks like Open & Agile Smart Cities (OASC) [74] and Eurocities [75]. They give cities a voice and help in developing solutions that can be replicated across cities. Especially, OASC has defined a set of Minimum Interoperability Mechanisms (MIMs) [76]. MIMs define a common basis to ensure interoperability of solutions:

- MIM 1 is called *OASC Context Information Management* and relates to the API that allows accessing to real-time context information. The recommended standard for this is NGSI-LD.
- MIM 2 is called *OASC Data Models* and refers to guidelines and a catalogue of common data models that enable interoperability for applications and systems among cities. There are several data models already proposed (e.g., SAREF [77] supported by ETSI, OneDM [78] supported by the Zigbee Alliance) but OASC seems to focus on the models by Smart Data Models initiative [79], which it recently joined.
- MIM3 is called *OASC Ecosystem Transactions Management* and refers to the Marketplace API that exposes functionality like catalogue, ordering and revenue management. Here, the TM Forum Open APIs [80] are proposed for building interoperable solutions.

Further MIMs like Personal Data Management and Fair Artificial Intelligence and Algorithms are currently under development.

An important role in this respect is played by open-source frameworks, which often provide reference implementations for standards. An example of such a framework is FIWARE [81] that is used by Santander introduced in Section 2.2. FIWARE is an open-source ecosystem managed by the FIWARE Foundation. It offers a set of open-source components that provide the functionality to assemble a smart city platform. Often the components implement standards like NGSI-LD or oneM2M. On this basis a smart city platform can be built and complemented with commercial components and applications where needed. Due to the general availability of the open-source components and by building on standards, it is nevertheless much easier to change providers or integrate existing deployments, avoiding vendor lock-in.

Last but not least, as pointed out in the context of OASC MIM 2, data models are critical for creating a global digital single market of interoperable and replicable (portable) smart solutions in multiple domains. Such models provide an essential element in the common technical ground needed for standards-based open innovation and procurement. One set of common data models is being developed by the Smart Data Models initiative6. The available models, organized in the form of openly accessible JSON Schemas, are meant to be widely adopted (de-facto standards) in a series of application domains (e.g., smart cities, smart agri-food, smart utilities, smart industry, …). In particular, besides the specific set of models that have been defined under the Smart City domain, other domains with dedicated models, for which Smart City applications are nowadays provided (cfr. Table 1 and Table 3), are Smart Environment, Smart Destination, Smart Water and Smart Energy.

### 3.4. Smart City Applications

Citizens living in smart cities expect a lot from their smart cities [82]. For example, smart cities are expected to leverage various ICT technologies to improve legacy city services and reduce the cost of living. Therefore, smart city services and their applications can extend into many diverse domains [83]. There is a smart city regulation in South Korea that classifies the smart city services into ten categories; administration, transportation, health, environment, security, disaster prevention, infrastructure management, education, culture & tourism, logistics, and employment [84].

There are many different ways to classify smart city services and applications. We have already shown this in Section 2. For example, we can see that existing smart city services in different cities (e.g., sizes, locations, regulations, and time) have significant differences in the strategy and solutions that they have adopted.

In this section, as summarized in Table 3, various smart city applications that run on top of smart city platforms using smart city APIs are introduced and classified based on our comprehensive review in Section 2. In particular, we classify smart city services into four high-level categories and provide several key smart city applications in each smart city application category.

- **Smart transportation:** This type of application is intended to establish a demand-responsive transportation system by installing transportation infrastructure suitable for the era of autonomous driving and providing consumer-oriented transportation services.
- **Smart environment:** This type of application introduces the latest smart water management technology for water resource management and water disaster prevention, and presents a water management leading model in waterfront cities.
- **Smart energy:** This type of application introduces new and renewable energy and establishes an energy demand management system to increase the energy independence of the city.
- **Smart security:** This type of application provides fast and accurate civil safety services by applying solutions using innovative technologies related to disaster and safety.

### 3.5. Artificial Intelligence and Big Data

The true value of the Internet of Things, especially applied to the smart city domain, lies in insights that can be gained from the sensor data and user-provided information collected on the IoT platform. The insights are the basis for enabling humans to take the right decisions or successfully automate important processes, all with the goals of increasing the operational efficiency of the city as well as improving the quality of life for the citizens. To get such insights, the raw data and low-level information constituting Big Data needs to be processed, using a variety of algorithms from the area of artificial intelligence, which are going to be discussed in the following.

Big Data is often characterized by five Vs–Volume, Velocity, Variety, Veracity and Value [85]. *Volume* relates to the amount of information. In a smart city a huge amount of information may be related to streams from video cameras or the creation of detailed 3D models, e.g., based on point clouds. *Velocity* refers to the speed at which information flows and needs to be handled. Again, this could be video streams or traffic information that needs to be processed in real-time. *Variety* relates to different types of information as well as different formats. For example, location can be provided in a variety of formats with different accuracies. *Veracity* refers to the quality of the data–how complete and accurate it is and to what extent is it a realistic representation of the situation, e.g., a temperature sensor may have good accuracy, but may not provide a correct representation of the room temperature if positioned closed to a window through which the sun is shining. The final V stands for *Value*, which represents the utility of the data, i.e., the insight that can be gained and what can be done with it.

As discussed in the context of IoT platforms in Section 3.3, it is important to agree on a common API for accessing and finding relevant city information as a minimum interoperability mechanism. However, with the variety and heterogeneity of information available in a smart city, not all information will be on the same abstraction level and on the same conceptual level. In particular, raw data may be required from which the high-level insights can be extracted. Depending on the Big Data Vs introduced above, different storage systems are required for handling the data and to enable the required processing. The storage of very heterogeneous data in different systems is often called a *data lake*. To be able to find the data in a data lake, key information can be extracted from the data lake and made available as homogenous information accessible through the IoT platform API, but linking to the complete original information, enabling to discover and further process the raw data.

The artificial intelligence-based algorithms for processing information can be categorized into data analytics, machine learning and deep learning [86]. For data analytics, there are the following four different approaches:

- **Descriptive Analytics** processes historic data and provides metrics and measures to summarize the information. It is often taken as an initial step followed by further analytics. For example, it can be used to find the peak of traffic congestion.
- **Diagnostic Analytics** after an issue has been detected, e.g., as a result of descriptive analytics, diagnostic analytics can be applied to attempt to understand the cause, e.g., rush hour traffic in combination with construction sites.
- **Predictive Analytics** can be used to forecast situations and events in the future. Historic information can be used to create a function that predicts a value, e.g., traffic congestion at a certain time on a weekday under certain weather conditions, which can be applied to predict the situation at a future point in time.
- **Prescriptive Analytics** goes one step further than predictive analytics as it tries to understand how different factors influence a situation. This can be used to take actions to improve the situation, e.g., adapt the traffic light scheduling to improve the traffic flow and reduce traffic congestion.

Machine learning approaches can be classified into four different categories [86]:

- **Supervised Learning** tries to learn how to map a set of input values to an output value. This requires labelled training data, i.e., data for which this mapping is given. After training, the resulting model is able to predict the output for new input data. For example, based on the movement characteristics of a user as extracted from a smart phone, the means of transport can be determined. Typical supervised learning techniques are Support Vector Machine (SVM), K-nearest Neighbour (KNN), Random Forest, Linear Regression (LR) and Decision Trees (DT).
- **Unsupervised Learning** tries to find clusters with closely related input values. For example, identifying tourists showing similar behaviour can be used for targeted advertising. In some cases, noise and outliers can also be found in a dataset. k-means and DBSCAN are typical examples of unsupervised learning techniques.
- **Reinforcement Learning** is an approach where a system can adapt its own behaviour in different ways, evaluating the results achieved. An improved result leads to a higher reward and the goal is to maximize the cumulative results. For example, reinforcement learning can be used to adapt traffic signal control, e.g., with the goal of minimizing queue length. Examples of reinforcement learning techniques are Q-learning and SARSA.
- **Deep Learning** represents a family of machine learning methods, where “*deep*” refers to the use of artificial neural networks with *multiple* layers. Deep learning is particularly suitable for applications with high data dimensionality and can cope with high volume and velocity of data. On the other hand, deep learning requires a large amount of training data. Typical deep learning techniques are Convolutional Neural Networks (CNN), Restricted Boltzman Machine and Deep Belief Networks.

To enable humans to make sense of the processing results, to grasp the insights gained and to take appropriate decisions as a result, data visualization is of paramount importance. The goal is to reveal patterns, show trends and find correlations in the underlying data. For example, line graphs are used to show how values like temperature and humidity change over time; Pie charts are useful to illustrate proportions, e.g., the age distribution of people frequenting a certain place. Heat maps can be used to show hotspots, e.g., areas specifically affected by bad air quality.

### 3.6. Security

A smart city is composed of diverse and complex digital IoT systems and devices. As IoT devices used in numerous services are being connected to various networks, security threats to all elements comprising the smart city pose potential risks to the entire smart city. In fact, attackers can exploit a vulnerability in the network to infect an IoT device with malware, and subsequently cause mass infection of other IoT devices and systems connected to the smart city [87].

Various security technologies have been developed for IoT-based smart cities. This section discusses secure network, authentication, secure booting, and data privacy technologies among the developed security technologies.

- **Secure booting:** When malware infects the boot sector of the system, it may not be detected by the security system present in the operating system. In particular, if malware infects a system on which a smart city IoT platform operates, a threat may be posed to the entire smart city infrastructure. To solve this problem, secure booting technology is implemented, thereby preemptively preventing operating systems and software that are not authorized. This security procedure technology serves as the first wall against various malicious attacks that can target a smart city. Secure booting technology checks the encrypted boot loader, kernel, and system software in authorized hardware. To this end, hardware is manufactured with authentication keys embedded in it. Secure booting technology checks such encrypted signatures before the operating system reads the kernel and system images, and only then the authenticated files are booted.
- **Secure networks:** IoT devices in a smart city use various network technologies to access the cloud-based IoT platform. To prevent exposing important information because of security threats occurring during data transmission, messages are encrypted and transmitted using encryption algorithms. For example, devices using Bluetooth versions prior to BLE 4.0/4.1 use encrypted authentication pairing, while BLE 4.2 devices use encrypted low-power security connection authentication feature. In the case of ZigBee, it defines security based on a 128-bit AES encryption algorithm, and uses three types of security keys (master, network, and link) for network security. The 3GPP 5G technology has additional security features to securely process network access volume increased by over ten times because of IoT devices. For this, the 3GPP uses international mobile subscriber identity (IMSI) encryption to protect subscriber information, security edge protection proxy (SEPP) that resolves the roaming domain security issue (Signaling System No. 7 [88]) and implements security between application layers among different carriers, and an integrated authentication framework feature that enables the use of the same authentication method for access to 3GPP and non-3GPP components.
- **Authentication and access control:** Devices and users connected to a smart city are managed using existing authentication and access control technologies. However, lightweight IoT-based devices are not powerful enough for using such technologies, hence, manual access control technologies are used. Various ways to resolve this issue are being proposed, including more detailed log and event monitoring, isolation of suspected devices, and role-based access control policies.
- **Data protection:** Ensuring the integrity of data constituting a smart city is an important task. If the integrity of key data is compromised, it may cause attacks on the overall smart city services and infrastructure. System settings information, log files, system libraries, and binary execution files are examples of data that must be protected in a smart city platform. Traditional cyclic redundancy check (CRC) and hash functions for SHA-2 and above are used to verify data integrity. In addition, smart cities inevitably collect personal information; in recent years, governments have been strengthening laws to protect personal information, enforcing systems handling personal information to follow such regulations. For example, the General Data Protection Regulations (GDPR) enacted in Europe in May 2018 puts strong restrictions on the processing, storage and usage of personal information and what is required for properly anonymizing information [89]. The regulations consider broad subjects spanning consent management, right to be deleted, and other personal information processing and management. Korea has also revised the existing personal information protection law to be compliant with the European GDPR, and other countries around the world are instituting laws to protect personal information and developing various supporting technologies for smart cities.

## 4. Open Challenges for IoT-Enabled Smart Cities

As population growth and urban concentration accelerate, various problems such as traffic congestion, energy and resource depletion, etc. are appearing all over the world, and countries are trying to solve these problems by using IoT-enabled smart city. Based on our experience and analysis in the previous sections, it is clear that smart city technologies can be a promising solution. However, it is clear that smart city technologies are not complete and still have many problems that must be overcome. For example, interoperability problems due to the absence of international standards, different understandings of smart cities, and exposure of personal information are major factors that hinder the sustainability of smart cities. In the past, the authors of this paper have accumulated many experiences on smart cities through the development of smart city services and platforms based on global IoT standards [22]. Based on these experiences and the research conducted in this paper, various challenges to be overcome by smart cities are derived.

In the following, we provide an outlook on the challenges that are currently being addressed as a precondition to making IoT-enabled Smart Cities a success.

### 4.1. Overcome Application Silos

Currently, most existing smart city applications have been developed as silo applications, often with a strong integration between applications and the underlying data sources, in particular sensors. This makes sharing and re-use of information difficult, as each application needs to be explicitly integrated with each existing information source and each change in technology, e.g., of underlying sensors or communication network requires an adaptation on the application side.

However, the true value of IoT will be achieved if more information becomes available and can be shared across applications and whole application domains [26,90]. It is often difficult enough to deploy a physical IoT infrastructure for one application, but it is not realistic to deploy the same or a similar infrastructure separately for the next application. By sharing infrastructure, synergies can be achieved and in some cases the deployment of infrastructure may only be feasible if it is shared.

To achieve this, a decoupling of information sources and applications is needed. On the technical side, this can be achieved using an IoT platform and the agreement on minimum interoperability mechanisms [76], e.g., as defined by the city network Open Agile Smart Cities (OASC).

At least as important for overcoming application silos as the technical side is the administrative and organizational side. Often applications are “owned” by city departments or organizations, which have paid for the infrastructure and wants to stay in control. Also, budgets are often assigned in an inflexible way, making sharing difficult. Thus, it is very important to reach a global understanding and define goals on a high level, e.g., for a whole city, and work towards achieving these goals across organizational units, often also overcoming personal rivalries in the process.

### 4.2. Evolve towards Flexible IoT Platforms

Currently, each smart city looks different with respect to infrastructure deployed, applications available, but often also the IoT platforms utilized. This makes it difficult to reuse anything developed for one city in another and constitutes an impediment with respect to creating a smart city market. Furthermore, it is not economically viable to develop custom-made platforms for each city [91].

As described in Section 3.3, a number of vendors have started to offer IoT platforms for smart cities, typically based on what has been developed for a pilot city. As a result, these platforms are often not a perfect fit for other cities and cities are also afraid of vendor lock-in, i.e., being restricted to this one vendor for any future additions regarding infrastructures and applications as everything has to be customized to the vendor’s platform.

The direction to go is to move from monolithic proprietary platforms to modular platforms based on standards (e.g., NGSI-LD [73], oneM2M [71], OMA Lightweight M2M [72]). It is especially important to agree on the interfaces and the modelling of information which OASC is defining as minimum interoperability mechanisms. Furthermore, costs can be reduced by relying on common open-source components as a basis, e.g., as provided by FIWARE. These can be complemented by proprietary vendor components. Nevertheless, the use of standards at the interface level prevents vendor lock-in.

### 4.3. Evolve towards Multi-Player, Cross-Organization IoT Platforms and Applications

Cities are not isolated islands, but often border on other cities and are part of districts, counties, regions and countries. For many applications, it is important that they do not stop working on city boundaries. It is not acceptable for citizens that they need different applications or have to reconfigure their applications if they cross a city boundary, as they may live in one city, work in another and spend their leisure time in a third. For example, there should be one application that helps them find a parking spot, ideally considering traffic, regardless of where they currently are.

This requires interworking of IoT platforms enabling the sharing of information, which can be realized by federating IoT platforms [92], partially making information available to this federation. For example, federated IoT platform architectures are explicitly supported in the NGSI-LD specification [73]. Thus, technically, such a setup is feasible, however again administrative and organizational issues have to be overcome as there are multiple players involved and the federated system has to work across organizations, which requires clarifying responsibilities and the allocation of budgets.

Given that federated IoT platform infrastructures exist that enable the sharing of information, marketplaces for information and applications can be created. Companies can offer their applications in the marketplace and while basic information may be available for free, premium information can be offered for a fee.

### 4.4. Business Opportunities Creation

While the smart city concept is typically bound to sustainability and efficiency by city administrators, digital transformation is opening new opportunities besides the optimization of the urban services that have traditionally been offered to its citizens (mobility, lighting, water distribution, waste management, etc.) [93]. The smart city has to be promoted as a scenario where new business models are created around the exchange of data and the services that arise from this sharing. For example, cities are becoming the playground for the sharing economy [94]. The concentration of economic activity and ubiquity of technology in cities facilitate the rapid, location-based exchange of services and products among citizens (P2P), among businesses (B2B), and businesses to the crowd.

Novel business concepts like the Commons Collaborative Economy [95] characterized by using data as the new key good for trading in the newly created digital marketplaces shall be able to:

(1) Favour peer-to-peer relations -in contrast to the traditionally hierarchical command and contractual relationships.
(2) Settle new value distribution and governance among the community of peers where profitability is not its main/unique driving force.
(3) Leverage privacy-aware public infrastructure that results in the (generally) open access provision of common resources that favour access, reproducibility and derivativeness.

However, the sharing of information is not trivial, especially if it is not foreseen in existing contracts. Thus, it is important to create awareness and consider this aspect when setting up contracts, especially when outsourcing activities. The monetization of information should become possible, however there is often a discrepancy between expectation and real value of information. Monetization has to be related to real value, e.g., in a similar way as it is the case for advertising on the web, where the use is measured and monetary value is directly related to it.

### 4.5. Achieve Transparency and Acceptance

While the smart cities paradigm has been, for long, shaped by sales pitches of larger technology vendors and system integrators, for this vision of self-sustainability and citizen’s quality of life improvement to become a reality, it is necessary to abandon early “smart city 1.0” examples such as Masdar or Songdo (with a technology- or marketing- driven approach rather than addressing operational and citizen needs) as well as to widen the pool of services supported by not only taking the perspective of the city authorities, but also allow addressing the specific needs of the citizens [96].

In this sense, even if there are significant cultural differences globally, it is necessary to set up the mechanisms to voice the citizen’s viewpoint in the current debate about IoT instrumentation. For smart city services to be able to make a real difference to citizens, and be well-perceived by all the stakeholders and actors involved in cities’ complex ecosystem, citizens must be empowered with the means to have a central role in the creation and design of such services [97].

The co-creation concept stands for the participatory development of applications and services collaboratively between several stakeholders. For this scenario to be enabled, platforms supporting smart cities have to provide a collection of intuitive tools and enablers that allow the specification and development of innovative applications. Additionally, it is necessary to counter the perception of loss of privacy to foster the platform to be fed by as many city assets as possible, and the information that they generate.

Another key aspect of the co-creation concept is its capacity to engage heterogeneous stakeholders in boosting smart city potential [98]. Consequently, smart city platforms need to provide enablers associated to the incentives and rewards of participating in the long-term sustainability of the whole platform and smart city ecosystem. In this sense, it is still necessary to develop a security-by-design approach increasing the transparency of all the IoT asset governance flow shifting from the current paradigm of discrete centralized trusted authorities to a paradigm of liquid and decentralized trust of the network as a whole. This shifting responds to the providers’ needs and to the consumers’ demands. The first because they are reluctant to share some datasets due to uncertainty of how and for what purposes the data is used. The latter because they require reassurance of the data’s quality.

### 4.6. Integration on Future Networks and Novel Computing Paradigms

As more smart city services are implemented, the number of devices connected to and operated on the network is growing exponentially. In addition, with the introduction of 5G network technology, various services such as smart city, smart factory, and smart car are being developed. Such diverse IoT services must support requirements such as high bandwidth and ultra-low latency [99].

Cloud computing methods provided by conventional service providers, however, must go through a central IoT platform to process various data generated by the devices. This has led to a rapid increase in the backhaul data traffic, inevitably slowing down service speeds. To solve this problem, extensive research is being conducted focusing on application of edge computing technology to reduce network load caused by traffic increase and to support IoT services from a relatively short distance, in turn, enabling the provisioning of ultra-low latency services [100].

For example, communication standardization organizations such as 3GPP and ETSI, as well as IoT standardization organizations such as oneM2M acknowledge the importance of edge computing standardization, cooperating with other institutions to develop standards and enhance compatibility. The Multi-access Edge Computing Industry Specification Group (MEC-ISG), a group in ETSI, is working on utilizing multi-access in edge computing and enabling compatibility with standards for network slicing provided by the 3GPP core network [100]. In addition to the virtualization of the network, virtualization and edge computing of IoT platform features are being conducted: One edge node will be able to host a 3GPP network, ETSI MEC edge computing, and ultra-low latency IoT service platform features dynamically to support various services.

### 4.7. Develop Resiliency to Support Mission-Critical Applications

In addition to the evolution of mobile broadband networks that the future generation of communication networks and services, so-called 5G, will be, it will also provide additional network and service capabilities of utmost importance for smart cities and, in particular, for IoT-enabled smart cities [99]. 5G is expected to be a fundamental enabler for the IoT by supporting the connection of a massive number of sensors, supporting devices and actuators with strict computational and power constraints. However, the modelling of these scenarios (mainly at the access network level), and which combination of technologies will be part of these future communication systems is still not yet fully developed.

Moreover, IoT-enabled smart cities will have to support real-time services that require very high reliability on the communication services [101]. Thus, there can be unquestionably zero down-time for mission critical services. In order to create robust situational awareness, it is of utmost importance that both the physical and communication infrastructure at the sensing layer and the fog/cloud platforms at the analysis and service layer have the security, availability and self-healing capabilities to support 99.99% Grade of Service under any circumstance.

There are no existing examples of mission/time critical applications for smart cities deployed at the moment since it is still necessary to develop solutions that increase the robustness and resiliency of the ecosystem for this kind of application to be supported with the required Grade of Service.

### 4.8. Privacy Regulations on Data in Different Countries

Because a smart city collects and utilizes massive amounts of data, it inevitably harbours the potential for personal information infringement. For example, sensors on IoT-based home appliances may be used to collect data from inside the home. Sensors in the interior of a smart car may also be used to observe the driving habits of the driver. Implementing data mining techniques on such information can lead to the identification of certain individuals. As such, governments are enacting strong laws on the protection of personal information to systematically protect personal information and prevent data-collecting service providers from abusing it [89].

For example, Europe enacted GDPR to regulate personal information protection and utilization. GDPR scrupulously defines the rights and obligations of personal information holders and processors, thereby strengthening the duties of companies that collect and manage data. As this law applies to all systems handling data, data-based smart cities must naturally comply with it.

Clauses of the GDPR [102] prescribe consent, right to be forgotten, pseudonymization, among others. These clauses must be reflected as system requirements and embedded into platform features, so that such clauses can be applied in a smart city platform. Doing so can enable various smart city services to process personal information in a manner compliant with GDPR. For example, consent management as defined by GDPR requires that personal information must be processed based on the consent of the data owner. However, existing smart city data platforms do not provide a feature that processes data owner consent. For this, the access authorization feature of the existing platforms should be expanded to implement the function of systematically acquiring user consent and classifying such data in the smart city system.

## 5. Conclusions

As can be seen from the examples described, a plethora of smart city applications and services have been developed. Many of them are at a proof-of-concept level, but more and more have already passed the pilot phase and have developed into production-level deployments. This shows that the minimum set of technology enablers is already available and is being deployed in cities and integrated with real-world public services.

However, often such services are being developed and deployed in application silos, limiting the value of the information that is being collected as re-use and sharing of this information is difficult. The use of IoT platforms enables such data accessibility and exchange, but the use of proprietary platforms that have been developed individually and in an isolated manner by large companies results in further restrictions as solutions cannot be replicated across cities relying on a different proprietary platform. As applications cannot be re-used, they have to be built from scratch or be adapted to a different platform, making developments expensive. The cities thus suffer from vendor lock-in, i.e., they cannot easily buy and integrate components or applications from a different vendor, but are stuck to the provider of their IoT platform.

The way out is to build on standards that are beginning to emerge. Smart city organizations have started to define standard-based minimum interoperability mechanisms. Another aspect is the use of open-source components that reduce costs and risks for cities while still giving room for standard-compliant commercial components. Building on standard-based minimum interoperability mechanisms is important for creating a real smart city market as applications, information and services can be used in many smart cities instead of having to be custom-made or require costly adaptations for each city. This reduces costs significantly and makes deployments on a larger scale viable.

As we have described, there are significant advances on the technical side that make smart cities feasible. Still, there are some technological challenges to overcome, mainly related to guaranteeing the necessary resiliency on data and networks to support mission-critical services (e.g., emergency response, critical infrastructures real-time monitoring and management, etc.). Integration of novel computing paradigms like fog computing and 5G and beyond networks in IoT scenarios unveils a promising path to address such application scenario. However, the biggest challenges may be on the administrative and organizational sides. Smart city deployments often require cooperation between different departments or even different organizations. This may require budgets to be readjusted, as there is the need for initial investments. These may be significantly offset by savings at a later point, but not necessarily where the initial investments have been made.

Moreover, technological or public administrations push cannot, on their own, untap the full value of the smart city paradigm. Surely, it can initiate the transition and settle the roots for all the stakeholders and actors involved in urban environments (e.g., citizens, companies, organizations, visitors, etc.) to become actively engaged in the smart city, not only as consumers of services or data that others provide, but also as producers of valuable information that would bring forward new services, thus contributing to a virtuous cycle with unforeseeable benefits. However, for this vision to become a reality, it is necessary to work on challenges of data security and self-sovereignty so that data producers are confident that their data is used for those, and only those purposes for which it was shared, and they are also appropriately and fairly rewarded for the value that their data has generated.

Finally, there is often resistance to change, especially if it does not bring immediate benefits to those who have to adapt. Making the city smart will lead to changes in the way the city operates. If new insights are gained, actions may have to be taken as a result. These have to be prepared and it requires buy-in from all the parties involved.

Nevertheless, smart cities in the long run will provide significant benefits as their operation becomes more efficient and the quality of life of their citizens improves. Thus, it is worthwhile to work on overcoming the obstacles, on the technical as well as the administrative and organizational side.

## Figures and Tables

**Table 1:** Summary of features from analysed cities.3. Key Technologies Developed.

City	Status	Motivations	IoT Infrastructure Deployed	Supported Services	IoT Platform	Communication Technologies	Start of Developments
Santander, Spain	Urban Lab + Smart Public Services	Research and Innovation testbed Progressive smartization of public services	12,000 sensors (testbed)	Parking Service Traffic Intensity Monitoring. Environmental Monitoring. Parks and gardens irrigation. Participatory Sensing Urban Waste Management. Water management Streetlight Management.Traffic Management	FIWARE	IEEE 802.15.4 LoRa Proprietary RF Cellular	2010
Busan, South Korea	Commercial smart city services are available	Sustainable and interoperable IoT-based smart city	Six IoT living labs for energy, factory, logistics, healthcare, urban regeneration and transportation	26 IoT services incl. Smart Parking, IoT mirror, VR smart tourism, Context-awareness safety warning, Smart street light, Smart drone safety service, Smart energy management, Smart traffic management service, Smart cross road safety	oneM2M	Cellular (3G/LTE/5G) LoRa IEEE 802.15.4	2016
Singapore	National level smart city. Commercial smart city services are available.	To resolve various future urban problems	Public WiFi access points Around 120 living labs	Virtual Singapore Urban planning Water usage management Smart street light Security warning buttonWaste management	Government supported proprietary platform.	WiFi	2014
Shenzen, China	City wide commercial services and platforms are available	A vision for using ICT to enhance public services, city management, and economic development	>400,000 NB-IoT enabled base stations	A city level health management portal Public surveillance system Water management Smart industry park Internet-coordinated manufacturing Sensors and actuators supporting street light automation	Proprietary developed with several ICT firms and universities	NB-IoT 5G	2018
Atlanta, US	Commercial smart city applications are available + Pilot-level services	Leverage a data-centric approach to improve mobility, public safety, and sustainability, for ultimately enhancing citizen well-being and fostering the economic growth	CCTV cameras 200 CityIQ sensor nodes (installed with various IoT sensors)	Mobility (e.g., North Av. Smart Corridor, Bike sharing) Safety (e.g., ShotSpotter, NotifyATL) Environment (e.g., Smart Neighbourhood, Smart trash bins)	Proprietary, developed by GE and Intel.	Cellular (3G, 4G) WiFi	2016
Amsterdam. Netherlands	Smart City umbrella organization supporting a variety of projects	Citizen-focused projects to improve quality of life, create sustainable growth and ensure efficient use of resources	iBeacons, Things Network with 46 gateways	Fuzzy navigation highlighting “hidden gems” MyNeighbour App supporting neighbourhood safety and sustainability Plastic-free rivers Car Pooling Smart Parking Open Data from different city departments	No central platform	LoRa, Bluetooth	2009
Sunshine Coast Region, Australia	Commercial smart city deployment offering a number of services	Improve city services, reduce resource consumption and improve the safety and quality of life of citizens. Support data-driven design and planning	>200 public WiFi access points, smart street lights, waste bin sensors, irrigation sensors, flood sensors	Sunshine Coast Council (SCC) App Smart Street with free public WiFi collecting data. Pedestrian and cyclist counters Networked LED street lighting Waste bin sensors Networked irrigation system Networked flood sensors	Proprietary, developed with Cisco and Telstra	WiFi	2016
Rio de Janeiro, Brazil	Commercial smart city services are available	Safety, security and disaster prevention; Smooth functioning of day-to-day operations of public services	500 surveillance cameras, GPS sensors on all garbage trucks,	Video surveillance of traffic Garbage truck fleet management Central 1746 (Participatory sensing)	Proprietary, developed with IBM	Cellular (3G, 4G)	2010

**Table 2:** Key features of IoT network technologies.

	NFC	BLE	Z-Wave	Zigbee	LoRa	SigFox	LTE-M	NB-IoT
Coverage	1~10 cm	3–30 m	30~100 m	30~100 m	3–15 km	5–25 km	~11 km	~15 km
Frequency	13.56 MHz	2.4 GHz	2.4 GHz	2.4 GHz	868–915 MHz	868–915 MHz	LTE in-band	LTE in-band
Data rate	106 ~ 424 kbps	1~3 Mbps	40~200 kbps	20/40/250 kbps	300 bps ~ 50 kbps	100 bps	~10 Mbps	~200 kbps
Payload size		358 bytes	64 bytes	127 bytes	256 bytes	12 bytes	-	-

**Table 3:** Smart city applications summary.

Category	Application	Description
Smart Transportation	Smart parking	Provides parking information in real time to improve the convenience of parking users and solve city parking difficulties.
	Smart tram	Improves public transportation efficiency by introducing an eco-friendly energy-based unmanned tram, and to build a smart transportation system that enables experience and promotion by applying advanced technologies such as digital tokens in a package format.
	Smart traffic control system	Improves traffic congestion by controlling signals by itself according to road conditions based on the analysis of traffic information collected in real time.
	Transportation sharing	Provides shared vehicle and bicycle services that can be used from departure to destination to eliminate blind spots in public transportation.
Smart Environment	Eco-filtering	Improves the quality of river water by creating an eco-friendly storage and treatment space with a natural purification function on the riverside to prevent the direct inflow of pollutants into the river.
	Fine dust management	Provides a fine dust forecast service with high spatial precision and prediction accuracy through IoT sensor data monitoring and learning, according to real-time environmental conditions.
	Smart filtration management	Small building type water purification facilities near consumers in the city centre are distributed and arranged to supply fresh, low-chlorine water to the home. Real-time water quality/quantity monitoring and remote monitoring control are provided.
	Water reuse	Developed as an alternative water resource from sewage treated water that has undergone an advanced treatment process, and is supplied as various necessary water (main transport water, maintenance water, washing water, etc.)
Smart Energy	Building energy management system	Provides a system that monitors building energy consumption in real time using IoT devices and automatically optimizes and controls and manages energy production and use.
	Energy efficient building	Uses eco-friendly and renewable energy to secure energy independence and provide an energy transaction system between individuals.
	Virtual power plant	Integrates the operation of small-scale distributed power facilities such as solar energy and household energy storage systems (ESS), and manages it as a single power plant.
Smart Security	Intelligent CCTV	Secures golden time by reducing human analysis errors and analysis time by real-time prediction of crime occurrence signs using visual intelligence technology and AI.
	School zone security	Secures commuting safety by linking information tracking the movement of students and vehicles around the school zone with vehicle information and introducing an accident prevention system.
	Disaster prediction	Prevents large-scale accidents by monitoring terrain changes and underground buried conditions with drones, satellites, and IoT sensors, and predicting AI-based ground subsidence.

## References

[1] Bairoch P. (1988). Cities and Economic Development: From the Dawn of History to the Present.

[2] United Nations, Department of Economic and Social Affairs “2018 Revision of World Urbanization Prospects”. https://www.un.org/development/desa/en/news/population/2018-revision-of-world-urbanization-prospects.html.

[3] Hoornweg D., Pope K. (2017). Population Predictions for the World’s Largest Cities in the 21st Century. Environ. Urban..

[4] Ochoa J.J., Tan Y., Qian Q.K., Shen L., Moreno E.L. (2018). Learning from Best Practices in Sustainable Urbanization. Habitat Int..

[5] Addanki S.C., Venkataraman H. (2017). Greening the Economy: A Review of Urban Sustainability Measures for Developing New Cities. Sustain. Cities Soc..

[6] Shahidehpour M., Li Z., Ganji M. (2018). Smart Cities for a Sustainable Urbanization: Illuminating the Need for Establishing Smart Urban Infrastructures. IEEE Electrif. Mag..

[7] Ménard A. How Can We Recognize the Real Power of the Internet of Things?. https://www.mckinsey.com/business-functions/mckinsey-digital/our-insights/how-can-we-recognize-the-real-power-of-the-internet-of-things#.

[8] D’Auria A., Tregua M., Vallejo-Martos M. (2018). Modern Conceptions of Cities as Smart and Sustainable and Their Commonalities. Sustainability.

[9] Alavi A.H., Jiao P., Buttlar W.G., Lajnef N. (2018). Internet of Things-Enabled Smart Cities: State-of-the-Art and Future Trends. Meas. J. Int. Meas. Confed..

[10] Gharaibeh A., Salahuddin M.A., Hussini S.J., Khreishah A., Khalil I., Guizani M., Al-Fuqaha A. (2017). Smart Cities: A Survey on Data Management, Security, and Enabling Technologies. IEEE Commun. Surv. Tutor..

[11] Heng T.M., Low L. (1993). Practioners’ Forum: The Intelligent City: Singapore Achieving the Next Lap. Technol. Anal. Strateg. Manag..

[12] Tariq M.A.U.R., Faumatu A., Hussein M., Shahid M.L.U.R., Muttil N. (2020). Smart City-Ranking of Major Australian Cities to Achieve a Smarter Future. Sustainability.

[13] Chamoso P., González-Briones A., Rodríguez S., Corchado J.M. (2018). Tendencies of Technologies and Platforms in Smart Cities: A State-of-the-Art Review. Wirel. Commun. Mob. Comput..

[14] Silva B.N., Khan M., Han K. (2018). Towards Sustainable Smart Cities: A Review of Trends, Architectures, Components, and Open Challenges in Smart Cities. Sustain. Cities Soc..

[15] Talari S., Shafie-khah M., Siano P., Loia V., Tommasetti A., Catalão J. (2017). A Review of Smart Cities Based on the Internet of Things Concept. Energies.

[16] Institute for Management Development, Singapore University for Technology and Design (SUTD) (2020). Smart City Index 2020.

[17] Berrone P., Ricart J.E. (2019). IESE Cities in Motion Index.

[18] Galache J.A., Santana J.R., Gutiérrez V., Sánchez L., Sotres P., Muñoz L. Towards Experimentation-Service Duality within a Smart City Scenario. Proceedings of the 9th Annual Conference on Wireless On-demand Network Systems and Services (WONS).

[19] Gutiérrez V., Galache J.A., Sańchez L., Munõz L., Hernández-Muñoz J.M., Fernandes J., Presser M. (2013). SmartSantander: Internet of Things Research and Innovation through Citizen Participation. Lecture Notes in Computer Science (Including Subseries Lecture Notes in Artificial Intelligence and Lecture Notes in Bioinformatics).

[20] Cirillo F., Solmaz G., Berz E.L., Bauer M., Cheng B., Kovacs E. (2020). A Standard-Based Open Source IoT Platform: FIWARE. IEEE Internet Things Mag..

[21] Cantera-Fonseca J.M., Galán-Márquez F., Jacobs T. FIWARE-NGSI v2 Specification. https://orioncontextbroker.docs.apiary.io/#.

[22] Sotres P., Lanza J., Sánchez L., Santana J.R., López C., Muñoz L. (2019). Breaking Vendors and City Locks through a Semantic-Enabled Global Interoperable Internet-of-Things System: A Smart Parking Case. Sensors.

[23] Chang F., Das D. (2020). Smart Nation Singapore: Developing Policies for a Citizen-Oriented Smart City Initiative. Developing National Urban Policies.

[24] Hu R. (2019). The State of Smart Cities in China: The Case of Shenzhen. Energies.

[25] City IQ platform. https://docs.cityiq.io.

[26] Martin-Caravaca S. How to Breaking Down Silos to Create a Better Smart City?. http://smartcitybrand.com/smart-city/how-to-breaking-down-silos-to-create-a-better-smart-city.

[27] Amsterdam Smart City. https://amsterdamsmartcity.com.

[28] Macpherson L. 8 Years On, Amsterdam Is Still Leading the Way as A Smart City. https://towardsdatascience.com/8-years-on-amsterdam-is-still-leading-the-way-as-a-smart-city-79bd91c7ac13.

[29] Bee Smart City Amsterdam Smart City: A World Leader in Smart City Development. https://hub.beesmart.city/city-portraits/smart-city-portrait-amsterdam.

[30] Sunshine Coast Council A Smarter Sunshine Coast, Smart City Brochure. https://d1j8a4bqwzee3.cloudfront.net/~/media/Corporate/Documents/SmartCities/SCIPBrochure.pdf?la=en.

[31] Sunshine Coast Council Smart City Program. https://www.sunshinecoast.qld.gov.au/smartcities.

[32] Central de Atendimento Rio. https://www.1746.rio/.

[33] Slaný V., Lučanský A., Koudelka P., Mareček J., Krčálová E., Martínek R. (2020). An Integrated IoT Architecture for Smart Metering Using Next Generation Sensor for Water Management Based on LoRaWAN Technology: A Pilot Study. Sensors.

[34] Abbas O., Abou Rjeily Y., Sadek M., Shahrour I. (2017). A Large-Scale Experimentation of the Smart Sewage System. Water Environ. J..

[35] Pasolini G., Toppan P., Zabini F., de Castro C., Andrisano O. (2019). Design, Deployment and Evolution of Heterogeneous Smart Public Lighting Systems. Appl. Sci..

[36] Saleem Y., Sotres P., Fricker S., Lopez de la Torre C., Crespi N., Lee G.M., Minerva R., Sanchez L. (2020). IoTRec: The IoT Recommender for Smart Parking System. IEEE Trans. Emerg. Top. Comput..

[37] Yuksel K., Kinet D., Chah K., Caucheteur C. (2020). Implementation of a Mobile Platform Based on Fiber Bragg Grating Sensors for Automotive Traffic Monitoring. Sensors.

[38] García-Domínguez A., Galvan-Tejada C.E., Zanella-Calzada L.A., Gamboa H., Galván-Tejada J.I., Celaya Padilla J.M., Luna-García H., Arceo-Olague J.G., Magallanes-Quintanar R. (2020). Deep Artificial Neural Network Based on Environmental Sound Data for the Generation of a Children Activity Classification Model. PeerJ Comput. Sci..

[39] Arroyo P., Herrero J., Suárez J., Lozano J. (2019). Wireless Sensor Network Combined with Cloud Computing for Air Quality Monitoring. Sensors.

[40] Huang H., Savkin A.V., Ding M., Huang C. (2019). Mobile Robots in Wireless Sensor Networks: A Survey on Tasks. Comput. Netw..

[41] Qi F., Zhu X., Mang G., Kadoch M., Li W. (2019). UAV Network and IoT in the Sky for Future Smart Cities. IEEE Netw..

[42] Anjomshoaa A., Duarte F., Rennings D., Matarazzo T.J., Desouza P., Ratti C. (2018). City Scanner: Building and Scheduling a Mobile Sensing Platform for Smart City Services. IEEE Internet Things J..

[43] Wang J., Jiang C., Zhang K., Quek T.Q.S., Ren Y., Hanzo L. (2018). Vehicular Sensing Networks in a Smart City: Principles, Technologies and Applications. IEEE Wirel. Commun..

[44] Lv B., Xu H., Wu J., Tian Y., Zhang Y., Zheng Y., Yuan C., Tian S. (2019). LiDAR-Enhanced Connected Infrastructures Sensing and Broadcasting High-Resolution Traffic Information Serving Smart Cities. IEEE Access.

[45] Guerrero-Ibáñez J., Zeadally S., Contreras-Castillo J. (2018). Sensor Technologies for Intelligent Transportation Systems. Sensors.

[46] Sanchez L., Muñoz L., Galache J.A., Sotres P., Santana J.R., Gutierrez V., Ramdhany R., Gluhak A., Krco S., Theodoridis E. (2014). SmartSantander: IoT Experimentation over a Smart City Testbed. Comput. Netw..

[47] Chen Y., Nakazawa J., Yonezawa T., Tokuda H. (2019). Cruisers: An Automotive Sensing Platform for Smart Cities Using Door-to-Door Garbage Collecting Trucks. Ad Hoc Netw..

[48] Burke A., Estrin D., Hansen M., Parker A., Ramanathan N., Reddy S., Srivastava M.B. (2006). Participatory Sensing. Proceedings of the First Workshop on World-Sensor-Web: Mobile Device Centric Sensory Networks and Applications (WSW 2006) at aACM SenSys 2006.

[49] Dutta P., Aoki P.M., Kumar N., Mainwaring A., Myers C., Willett W., Woodruff A. (2009). Common Sense—Participatory Urban Sensing Using a Network of Handheld Air Quality Monitors. Proceedings of the 7th ACM Conference on Embedded Networked Sensor Systems, SenSys 2009.

[50] Campbell A.T., Eisenman S.B., Lane N.D., Miluzzo E., Peterson R.A. (2006). People-Centric Urban Sensing. Proceedings of the ACM International Conference Proceeding Series.

[51] Ma H., Zhao D., Yuan P. (2014). Opportunities in Mobile Crowd Sensing. IEEE Commun. Mag..

[52] Sanchez L., Gutierrez V., Galache J.A., Sotres P., Santana J.R., Muñoz L. (2014). Engaging Individuals in the Smart City Paradigm: Participatory Sensing and Augmented Reality. Interdiscip. Stud. J..

[53] Mohan P., Padmanabhan V.N., Ramjee R. (2008). Nericell: Rich Monitoring of Road and Traffic Conditions Using Mobile Smartphones. Proceedings of the SenSys’08—6th ACM Conference on Embedded Networked Sensor Systems.

[54] Liu X., Song Z., Ngai E., Ma J., Wang W. (2015). PM2:5 Monitoring Using Images from Smartphones in Participatory Sensing. Proceedings of the IEEE INFOCOM Workshops.

[55] Montenegro G., Kushalnagar N., Hui J., Culler D. (2007). Transmission of IPv6 Packets over IEEE 802.15.4 Networks.

[56] DigiMesh Products. https://www.digi.com/products/browse/digimesh.

[57] Z-Wave Technology. https://www.z-wave.com/.

[58] Zigbee Alliance, WPAN Industry Group The Industry Group Responsible for the ZigBee Standard and Certification. http://www.zigbee.org.

[59] Bluetooth Core Specification Working Group Bluetooth Core Specification. https://www.bluetooth.com/specifications/bluetooth-core-specification/.

[60] Haartsen J.C. (2000). Bluetooth Radio System. IEEE Pers. Commun..

[61] ECMA International (2013). ECMA-340. Near Field Communication-Interface and Protocol (NFCIP-1).

[62] Sornin N., Luis M., Eirich T., Kramp T., Hersent O. (2015). LoRaWAN Specification.

[63] Sigfox Device Radio Specifications. https://build.sigfox.com/sigfox-device-radio-specifications.

[64] (2016). 3GPP. Evolved Universal Terrestrial Radio Access (E-UTRA); NB-IOT; Technical Report for BS and UE Radio Transmission and Reception.

[65] 3GPP Release 13. https://www.3gpp.org/release-13.

[66] Mekki K., Bajic E., Chaxel F., Meyer F. (2019). A Comparative Study of LPWAN Technologies for Large-Scale IoT Deployment. ICT Express.

[67] Sigfox Coverage Map. www.sigfox.com/en/coverage.

[68] Internet of Things (IoT) on IBM Cloud. https://www.ibm.com/cloud/internet-of-things.

[69] Microsoft Azure IoT. https://azure.microsoft.com/en-us/overview/iot/.

[70] Google Cloud IoT Solutions. https://cloud.google.com/solutions/iot.

[71] OneM2M. https://onem2m.org/.

[72] OMA Lightweight M2M. https://omaspecworks.org/what-is-oma-specworks/iot/lightweight-m2m-lwm2m/.

[73] ETSI ISG CIM. NGSI-LD. https://www.etsi.org/committee/cim.

[74] Open and Agile Smart Cities (OASC). https://oascities.org/.

[75] Eurocities. https://eurocities.eu/.

[76] OASC Minimum Interoperability Mechanisms, Open & Agile Smart Cities. https://oascities.org/minimal-interoperability-mechanisms/.

[77] Smart Applications REFerence Ontology, and Extensions. https://saref.etsi.org/.

[78] One Data Model. https://onedm.org/.

[79] Smart Data Models initiative. https://smartdatamodels.org/.

[80] TM Forum Open APIs. https://projects.tmforum.org/wiki/display/API/Open+API+Table.

[81] FIWARE Foundation. https://www.fiware.org.

[82] Willems J., van den Bergh J., Viaene S. (2017). Smart City Projects and Citizen Participation: The Case of London. Public Sector Management in a Globalized World.

[83] Gaur A., Scotney B., Parr G., McClean S. (2015). Smart City Architecture and Its Applications Based on IoT. Procedia Computer Science.

[84] Mehmood Y., Ahmad F., Yaqoob I., Adnane A., Imran M., Guizani S. (2017). Internet-of-Things-Based Smart Cities: Recent Advances and Challenges. IEEE Commun. Mag..

[85] Younas M. (2019). Research Challenges of Big Data. Serv. Oriented Comput. Appl..

[86] Habibzadeh H., Kaptan C., Soyata T., Kantarci B., Boukerche A. (2019). Smart City System Design: A Comprehensive Study of the Application and Data Planes. ACM Comput. Surv..

[87] Eckhoff D., Wagner I. (2018). Privacy in the Smart City—Applications, Technologies, Challenges, and Solutions. IEEE Commun. Surv. Tutor..

[88] Russell T. (2006). Signaling System# 7.

[89] Seo J., Kim K., Park M., Park M., Lee K. An Analysis of Economic Impact on IoT under GDPR. Proceedings of the International Conference on Information and Communication Technology Convergence: ICT Convergence Technologies Leading the Fourth Industrial Revolution, ICTC 2017.

[90] Soe R.-M. (2018). Smart Cities: From Silos to Cross-Border Approach. Int. J. E Plan. Res..

[91] Upton N., Hewlett Packard Enterprise Why the Internet of Things Demands a Flexible Platform?. https://internetofthingsagenda.techtarget.com/blog/IoT-Agenda/Why-the-internet-of-things-demands-a-flexible-platform.

[92] Santolalla O. Why Smart Cities Services Need Federated Access. https://www.ubisecure.com/stories/smart-cities/.

[93] Appio F.P., Lima M., Paroutis S. (2019). Understanding Smart Cities: Innovation Ecosystems, Technological Advancements, and Societal Challenges. Technol. Forecast. Soc. Chang..

[94] McLaren D., Agyeman J. (2015). Sharing Cities: A Case for Truly Smart and Sustainable Cities.

[95] Fuster-Morell M., Carballa-Smichowski B., Smorto G., Espelt R., Imperatore P., Rebordosa M., Rocas M., Rodríguez N., Senabre E., Ciurcina M. Multidisciplinary Framework on Commons Collaborative Economy; Decode Project. https://decodeproject.eu/publications/multidisciplinary-framework-commons-collaborative-economy.

[96] Sepasgozar S.M.E., Hawken S., Sargolzaei S., Foroozanfa M. (2019). Implementing Citizen Centric Technology in Developing Smart Cities: A Model for Predicting the Acceptance of Urban Technologies. Technol. Forecast. Soc. Chang..

[97] Van Kranenburg R., Stembert N., Moreno M.V., Skarmeta A.F., López C., Elicegui I., Sánchez L. (2014). Co-creation as the Key to a Public, Thriving, Inclusive and Meaningful EU IoT. Ubiquitous Computing and Ambient Intelligence. Personalisation and User Adapted Services.

[98] Gutiérrez V., Amaxilatis D., Mylonas G., Muñoz L. (2018). Empowering Citizens Toward the Co-Creation of Sustainable Cities. IEEE Internet Things J..

[99] Rao S.K., Prasad R. (2018). Impact of 5G Technologies on Smart City Implementation. Wirel. Pers. Commun..

[100] Sabella D., Vaillant A., Kuure P., Rauschenbach U., Giust F. (2016). Mobile-Edge Computing Architecture: The Role of MEC in the Internet of Things. IEEE Consum. Electron. Mag..

[101] Skarin P., Tarneberg W., Arzen K.E., Kihl M. Towards Mission-Critical Control at the Edge and over 5G. Proceedings of the IEEE International Conference on Edge Computing, EDGE 2018.

[102] Badii C., Bellini P., Difino A., Nesi P. (2020). Smart City IoT Platform Respecting GDPR Privacy and Security Aspects. IEEE Access.

